# Improving the accuracy of expression data analysis in time course experiments using resampling

**DOI:** 10.1186/s12859-014-0352-8

**Published:** 2014-10-25

**Authors:** Wencke Walter, Bernd Striberny, Emmanuel Gaquerel, Ian T Baldwin, Sang-Gyu Kim, Ines Heiland

**Affiliations:** Department of Molecular Ecology, Max Planck Institute for Chemical Ecology, Hans-Knöll-Straße 8, D-07745 Jena, Germany; Department of Arctic and Marine Biology, UiT The Arctic University of Norway, Naturfagbygget, Dramsvegen 201, 9037 Tromsø, Norway; Center for Organismal Studies, University of Heidelberg, Im Neuenheimer Feld 360, 69120 Heidelberg, Germany; Center for Genome Engineering, Institute for Basic Science, Gwanak-ro 1, Gwanak-gu, Seoul, 151-747 South Korea

**Keywords:** Resampling, Gene expression data, ARSER, Biological replicates, Circadian rhythms, HAYSTACK

## Abstract

**Background:**

As time series experiments in higher eukaryotes usually obtain data from different individuals collected at the different time points, a time series sample itself is not equivalent to a true biological replicate but is, rather, a combination of several biological replicates. The analysis of expression data derived from a time series sample is therefore often performed with a low number of replicates due to budget limitations or limitations in sample availability. In addition, most algorithms developed to identify specific patterns in time series dataset do not consider biological variation in samples collected at the same conditions.

**Results:**

Using artificial time course datasets, we show that resampling considerably improves the accuracy of transcripts identified as rhythmic. In particular, the number of false positives can be greatly reduced while at the same time the number of true positives can be maintained in the range of other methods currently used to determine rhythmically expressed genes.

**Conclusions:**

The resampling approach described here therefore increases the accuracy of time series expression data analysis and furthermore emphasizes the importance of biological replicates in identifying oscillating genes. Resampling can be used for any time series expression dataset as long as the samples are acquired from independent individuals at each time point.

**Electronic supplementary material:**

The online version of this article (doi:10.1186/s12859-014-0352-8) contains supplementary material, which is available to authorized users.

## Background

Even with decreasing costs for sequencing and microarray experiments, time series experiments are still expensive and require a large number of samples. Thus, most time series currently have a very limited number of biological replicates. This makes it difficult to identify genes that truly show time-dependent expression patterns (true positives) and genes that just seem to have similar patterns due to biological variance (false positives). The biological variance is likely to be relatively high, especially when samples are collected from higher eukaryotes, because animals and plants are usually sampled from different individuals to avoid perturbation artifacts during sampling. Thus, in most time course experiments, the samples at each time point are usually from different individuals, resulting in a high biological variance among samples. This is the main reason why sufficient numbers of replicates are necessary. Lee *et al*. proposed that three replicates are sufficient, but this number also depends on the type of experiment [1-4]. However, the importance of biological replicates is often neglected in time series experiments, especially when circadian rhythms in gene expression are examined using transcriptomics datasets.

Many organisms have an endogenous clock, known as a circadian clock, to coordinate daily activities. The output of the circadian clock has the period of approximately 24 h; for example, the body temperature and sleep-wake cycle in humans, leaf movement in *Mimosa*, and flower opening in night-blooming jasmine all show 24 h diurnal rhythms under both light/dark and approx. 24 h rhythms under constant conditions [5-7]. Although the molecular components of circadian clocks are not conserved between animals and plants, negative and positive feedback loops in transcriptional and post-translational levels are the core system of circadian clocks in both animals and plants [8]. These multiple interlocked feedback loops confer stability and protection from stochastic perturbations on the complexity of the circadian system [9,10]. To understand this complex network on a transcriptional level, time series microarrays have been frequently used to examine the oscillation of genes on a genomic scale [11,12].

Diurnal rhythms in transcript accumulation can be described in mathematical terms, including period, phase, and amplitude [10]. There are several different algorithms that can be used to calculate these parameters from real data; they can furthermore be applied to identify oscillating genes in microarray or RNA-sequencing data. From the algorithms available we selected ARSER [13], HAYSTACK [14] as well as the algorithms implemented in BIODARE (http://www.biodare.ed.ac.uk/) [15,16]. ARSER was selected as it has been shown to outperform earlier available algorithms such as COSOPT and Fisher’s G-Test [13]. The BIODARE platform is not originally designed for the analysis of gene expression data as the maximal list length of datasets that can be submitted is limited to 2500. Thus, gene expression data has to be split into multiple datasets. Nevertheless BIODARE has the advantage of providing 6 additional different algorithms for the analysis.

Using these algorithms we show the influence of replicates and resampling on the accuracy of predictions of rhythmically expressed genes. Although we perform the analysis to identify oscillating genes in circadian expression datasets, the resampling method can be similarly used to improve the detection of other time dependent expression patterns as long as the samples are collected from different individuals at the specific time points.

## Results and discussion

The determination of oscillating genes is a binary classification. There are only two possible outcomes: either a gene is rhythmically expressed or it is not. The accuracy of this classification can be estimated by a confusion matrix. There are four fundamental members of the matrix: true positives (expression profiles correctly classified as periodic), false negatives (expression profiles incorrectly classified as non-periodic), true negatives (expression profiles correctly classified as non-periodic), and false positives (expression profiles incorrectly classified as periodic). As the number of true negatives and false negatives can be directly calculated from the total number of oscillating and non-oscillating genes and the number of true- and false positive genes identified, we only analyzed true- and false-positives in our calculations. The total number of oscillating and non-oscillating genes was set to 8400 in our simulated datasets (see Methods section for details).

To calculate the performance of ARSER, HAYSTACK and the algorithms implemented in BIODARE, we simulated different conditions and wave forms for oscillating transcripts. To do this we used three different simulation procedures.

To simulate entrained, synchronized oscillations all simulations were done with a fixed period ranging from 22 to 28 h (LD-dataset). In contrast, the differences in free running period between different individuals under constant conditions were simulated by generating a dataset that contained 36 time courses that differed in period according to published standard deviations for individual cells [17] (LL-dataset). In addition we generated a time course based on a published ordinary equation model of the mammalian circadian model [18] (ODE-dataset) (see Methods section for details).

**Figure 1:**
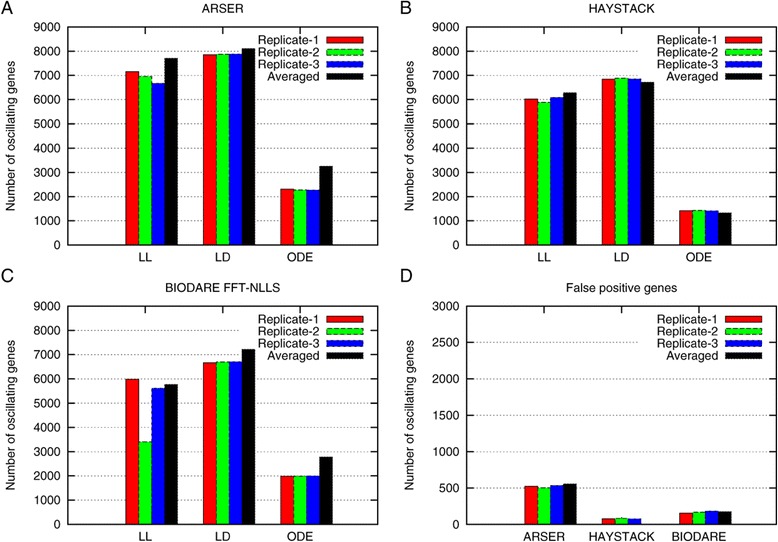
**Identification of oscillating transcripts in each replicate and the average dataset.** True positives **(A-** **C)** and false positives **(D)** for LD. LL and ODE-based time courses using ARSER, HAYSTACK or BIODARE FFT-NLLS The results are displayed for each individual replicate and the average dataset. The replicates were generated as described in the Methods section.

For each simulation procedure 36 time courses were initially calculated, corresponding to the common experimental time courses for gene expression analysis in the literature that resample 2-day time courses with 4 h sampling intervals and 3 replicates. From these initial time courses we generated the initial dataset (3 replicate time courses) by randomly selecting one time point from each simulated time course. These initial datasets were in addition averaged to generate a fourth, averaged time course. True and false positives were then calculated for ARSER, HAYSTACK and using BIODARE. From BIODARE we initially tested all implemented algorithms but found that FFT-NLLS was performing best, confirming the observations form Zielinski *et al*. [15,16]. We therefore only present the results from this BIODARE algorithm (Figure 1). In comparison ARSER detects the largest number of true positives but at the cost of a relatively high number of false positives.

In the averaged time courses the number of true positives detected by ARSER is in most cases slightly higher than in the individual replicates but this again comes at the cost of a higher number of false positives. HAYSTACK and BIODARE FFT-NLLS show similar performance but HAYSTACK has more problems to detect oscillating genes in ODE-based simulations. As detected false positive genes can be experimentally quite costly in follow up studies, we wanted to improve the accuracy of the prediction without increasing the number of replicates or time points required as this too would be experimentally costly if not infeasible.

**Figure 2:**
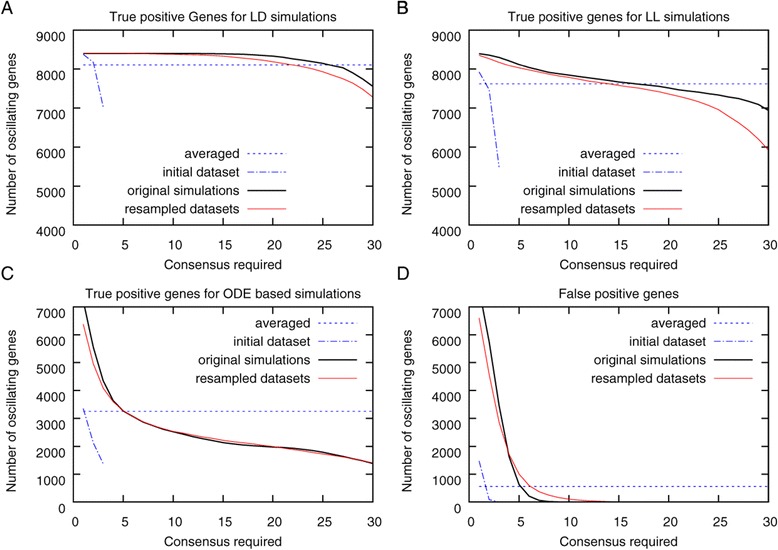
**Performance evaluation of the resampling method to identify oscillating transcripts.** For LL-, LD- and ODE-based time courses 36 time courses were simulated (original simulations). From these original simulations 3 replicates (initial dataset) for each type of time course were generated by randomly selecting one time point from each of the original simulations to mimic experimental sampling procedures. To generate the averaged dataset, the expression values of the 3 replicates at each time point were averaged. The initial datasets were furthermore used to generate 36 resampled datasets by random sampling at each time point. All datasets were analyzed with ARSER and true positives **(A-** **C)** and false positives **(D)** were calculated requiring increasing consensus between the datasets (see Methods for details). A consensus of 10 thereby means that a gene is found in at least 10 different resampled or originally simulated datasets. For the averaged time course the true and false positives calculated are displayed as a line for comparison.

**Figure 3:**
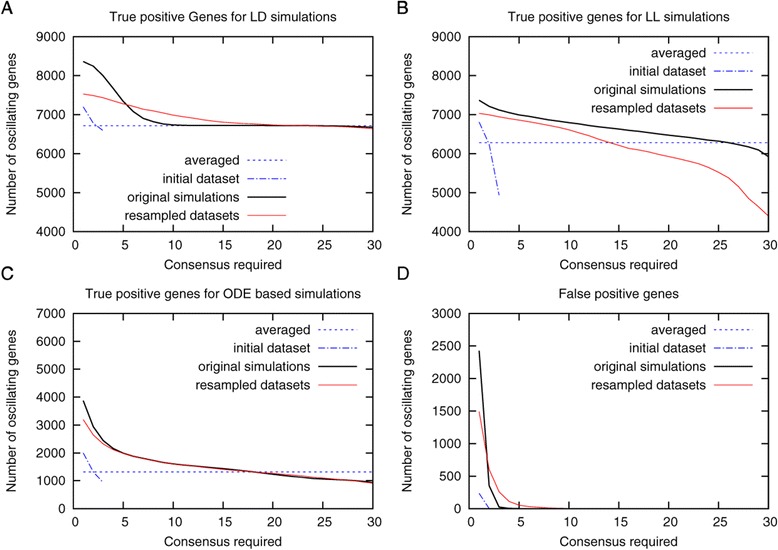
**Analysis of resampled time courses with HAYSTACK.** For LL-, LD- and ODE-based time courses 36 time courses were simulated (original simulations). From these original simulations 3 replicates (initial dataset) for each type of time course were generated by randomly selecting from each of the original simulations one time point to mimic experimental sampling procedures. To generate the averaged dataset, the expression values of the 3 replicates at each time point were averaged. The initial datasets were furthermore used to generate 36 resampled datasets by random sampling at each time point. All datasets were analyzed with HAYSTACK and true positives **(A**-**C)** and false positives **(D)** were calculated requiring increasing consensus between the datasets (see Methods for details). A consensus of 10 thereby means that a gene is found at least in 10 different resampled or originally simulated datasets. For the averaged time course the true and false positives calculated are displayed as a line for comparison.

We hypothesized that transcripts identified several times in resampled datasets contain more true positive and fewer false positive transcripts. To test this hypothesis, we generated 36 resampled datasets and identified oscillating genes by ARSER and HAYSTACK algorithms in each resampled dataset. Subsequently, we calculated the consensus of detected oscillating genes in these 36 resampled datasets. A consensus of 10 means that the genes were detected in at least 10 out of the 36 resampled datasets. The consensus graphs for the analysis performed with ARSER and HAYSTACK are shown in Figures 2 and 3, respectively. We compared the number of true and false positives to the number of true and false positives found in the averaged dataset, as well as to the consensus between the initial datasets and the initial simulations. The initial simulations represent the ideal situation that samples could be retrieved from the same individual, this is, however, not possible for gene expression analysis in most cases. It nevertheless represents the maximal detectable number of true positive transcripts in a noisy dataset. As can be seen from Figure 2, up to a required consensus between 15 datasets, the resampled datasets show a larger number of true positives compared to average and the overlap between initial datasets. To acquire the same consensus the number of false positives is 8 of 8400. A similar number of false positives is found if a full overlap between the 3 initial datasets is required. The number of true positives for the latter is, however, much lower for all types of simulations. We can therefore conclude that the resampling of datasets increases the number of true positive oscillating transcripts detected in a dataset without increasing the number of false positives compared to the initial replicates. Except when very low consensus is required (less than 7 for resampled dataset and 2 for initial datasets), the number of false positives detected with ARSER is always higher for averaged datasets, and hence not well suited to reliably identify oscillating genes.

**Figure 4:**
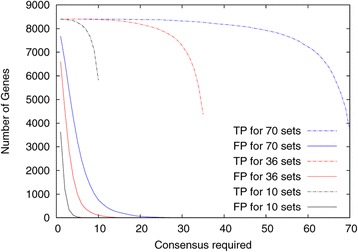
**Dependency of the analysis on the number of resampled dataset.** 3 initial datasets were generated as described in Figure 2 and in the Methods section and from these either 10, 36 or 70 resampled datasets were generated by random resampling and the number of true (TP) and false positive (FP) transcripts calculated. The detection of oscillating genes was performed with ARSER.

**Figure 5:**
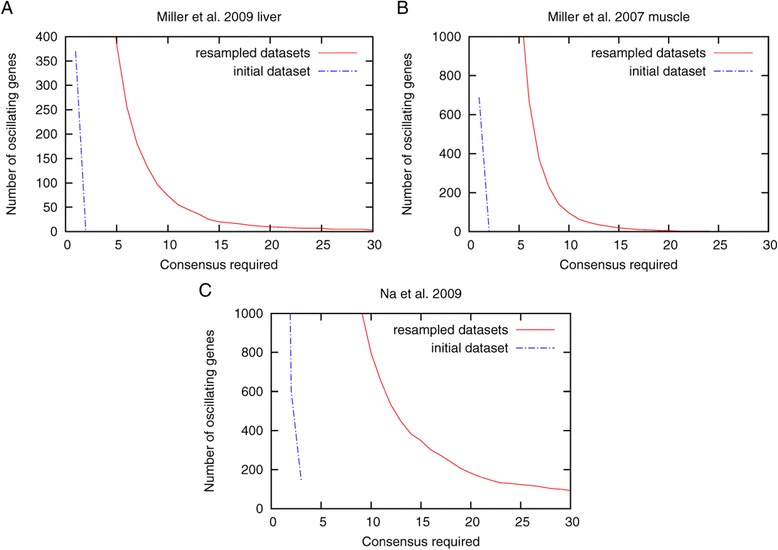
**Analysis of published expression data.** We reanalyzed 2 published circadian datasets with 4 hour sampling intervals and 12 time points using ARSER. The dataset by Miller *et al*. [19] contained only two replicates each for two mouse tissues (liver **(A)** and muscle **(B)**). The dataset from Na *et al*. [20] **(C)** contained 3 replicates.

We next analyzed the influence of the number of resampled datasets on the detection of true and false positive oscillating transcripts. As can be seen in Figure 4, higher consensus is required for a higher number of resampled datasets but the consensus range in which no false positives are found and in which the number of true positives remains high, is larger when a larger number of randomized datasets are analyzed. For the analysis of real data we therefore chose to generate 70 resampled datasets. Unfortunately there are very few circadian datasets available with sufficient replicates and time points. We found one study with two replicates performed in two different mouse tissues (liver and muscle) [19] and one other mouse study with 3 replicates [20]. The overall number of oscillating genes found in the dataset from Miller *et al*. [19] is similar to that reported in the original article. The overlap, however, was not analyzed in the original work and we only found 2 and 3 transcripts, respectively (Figure 5A and B) in both replicates. Using our resampling approach we identified 74 and 96 genes when requiring consensus between at least 10 sets and 10 and 5 transcripts, respectively, if a consensus of 20 was required.

For the dataset from Na *et al*. [19] 147 transcripts were found in all 3 initial replicates (Figure 5C). In our resampled dataset 796 transcripts were identified as oscillating when we require a consensus of 10, 183 genes remain if we require a consensus of 20.

**Figure 6:**
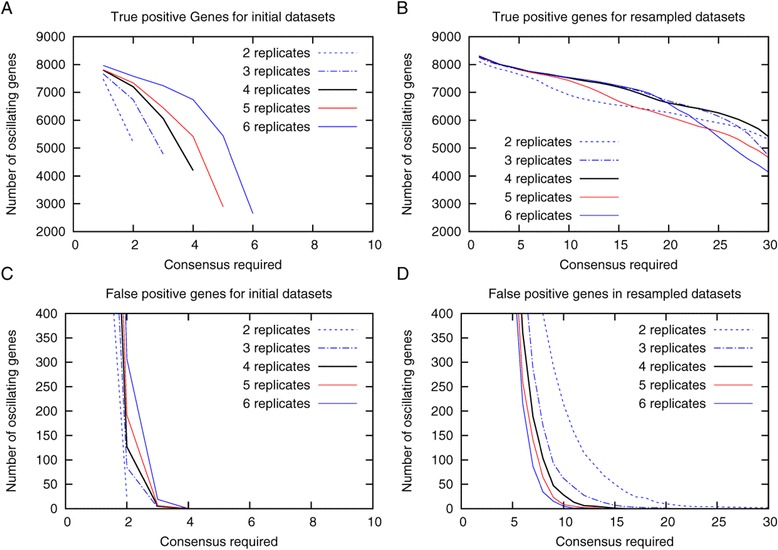
**Impact of the number of replicates on the accuracy.** From 72 original simulations, either 2,3,4,5, or 6 replicates were generated by random sampling** (A** **and** **C)** and analyzed using the ARSER algorithm as described in the Methods section. The initial datasets were then used to generate 36 resampled datasets **(B** **and** **D)** as described in Figure 2 and the Methods section. The number of true positives **(A and** **B)** and false positives **(C and** **D)** for different consensus required is shown.

As the study by Na *et al*. resulted in a larger number of oscillating transcripts we used our simulated LL datasets to analyze how the number of replicates influences the number of true and false negatives and thus the accuracy of the detection of oscillating transcripts. To do so we initially simulated 72 datasets. Those were used to generate the different numbers of initial replicated datasets. The analysis showed that the number of oscillating transcripts detected for a full overlap between all replicates is decreasing with the number of replicates (Figure 6A) with increasing consensus required. But starting from a required consensus of 4, false positives were no longer detected in the initial datasets (Figure 6C), thus a consensus of 4 is sufficient to accurately detect oscillating transcripts for initial datasets. Taken this into account the amount of true positives transcripts is higher for higher numbers of replicates as would be expected. Looking at the resampled datasets we see that with increasing number of replicates lower consensus is required to avoid detection of false positives, emphasizing the importance of replicates for the detection of circadian regulated transcripts.

## Conclusions

In this analysis, we conclude that in comparison to single replicates and averaged datasets, our resampling method improves the detection of oscillating transcripts without increasing the number of false positives. The resampling method particularly outperformed the average method to reduce the number of false positive transcripts. Furthermore, the resampling method shows that biological replicates are important to accurately identify true oscillating transcripts using time series gene expression datasets, and that the average method may result in a large number of false positives. To reliably identify oscillating transcripts, resampled datasets should be generated from at least 3 experimental samples per time point.

## Methods

### Simulated time series

As there is no way to determine whether an algorithm can distinguish true oscillating transcripts (true positives) from non-oscillating transcripts (false positives) in a real gene expression dataset, we generated artificial time series to analyze the performance of different algorithms. The artificially generated time series contained the expression values of 8400 transcripts. To generate periodic patterns for synchronized datasets (LD dataset), we used the formula by Yang and Su [13]. Thus the model is defined by:

1 $$ {x}_t=SNR\kern0.1em \cdotp \kern0.1em 2 cos\frac{2\pi }{\tau}\left(t-\varphi \right)+{\varepsilon}_t $$

where SNR = 2 is the signal-to-noise ratio; τ is the period in the range of 22 and 28 hours; ϕ is phase (0-28 h with 0.1 h intervals); and ε_t_ is the normally distributed noise term (mean =0 standard deviation =1).

Desynchronizing individuals under constant condition (for example constant light (LL)) were simulated using the above formula but with a fixed period that was randomly selected for each of the 36 initially simulated time courses. The periods were normally distributed with a mean of 25 hours and a standard deviation of 3 h according to published experimental data [9].

For more realistic circadian simulations we used the ODE-model of the mammalian circadian oscillators by Leloup *et al*. [18]. We first generated time courses for all variable model species and then generated phase shifted copies thereof. Phase shifts had 0.1 h intervals. From these time courses, datasets with 4 h sampling intervals were generated. Normally distributed white noise was added as for the cosine wave simulations. To simulate non-periodic time series, we used normally distributed white noise with the same mean and standard deviation as above.

The simulations described above were repeated 36 times for each type of data. If not described otherwise we generated 3 initial datasets from these time courses by randomly selecting once from each original simulation to generate a new 4 hour interval time course. This mimics the sampling procedure from different individuals in real experiments.

Python scripts used to generate time series and initial datasets are provided as Additional file 1.

### ARSER, HAYSTACK and BIODARE

Recently, Yang and Su developed the algorithm ARSER, which combines frequency domain and time domain analyses [13]. The algorithm first removes any linear trend from time series data (data preprocessing), and then the period is determined by AR spectral analysis (period detection). Because the period can differ from 24 h depending on the experimental conditions, the algorithm takes a range from 20 h to 28 h into account. With each period, ARSER employs harmonic regression to determine the four cyclic parameters: period, amplitude, mean level, and phase (rhythm modeling). Finally, false discovery rate (FDR) *q*-values are calculated for multiple comparisons and the output was filtered and only those transcripts with a *q*-value greater than 0.05 were consider in the analysis.

To exclude the possibility that our results depend on the chosen algorithm, the analysis was repeated with the HAYSTACK algorithm [21] and the FFT-NLLS algorithm implemented in BIODARE [15,16]. HAYSTACK was designed to find periodic patterns in any large-scale dataset representing at least three data points. The web version and 120 cycling patterns are available at http://haystack.mocklerlab.org/. The HAYSTACK algorithm compares gene expression profiles with predefined cycling patterns. Different cutoffs are used to detect oscillating patterns in gene expression. The most important parameter is the correlation coefficient. The higher value means a higher correlation between the experimental data and the predefined models. A coefficient of +1 indicates perfect positive correlation. Other cutoff values are the fold change and *p-*value, and these values are used to achieve statistical significance. The HAYSTACK algorithm searches for at least six different patterns, including “asymmetric,” “rigid,” “spike,” “cosine,” “sine,” and “box-like” patterns. The models that most successfully identify rhythmically expressed genes are “cosine” and “spike.”

BIODARE and the implemented algorithms are described elsewhere [15]. Shortly, FFT-NLLS (Fast Fourier Transform - Non-Linear Least Square) is a curve fitting method which models a sum of cosine functions and calculates confidence levels for period, phase and amplitude. The BIODARE FFT-NLLS algorithm detection was limited to period range from 20 to 28 h to match the period range of ARSER. Linear detrending was applied.

### Resampling

The artificially generated time series dataset consists of 12 time points at 4 h sampling intervals, representing 48 h of observation. To generate resampled datasets, expression values of each gene were randomly selected from (if not stated otherwise) three initial replicate time series, and the values were combined to generate the new resampled dataset. Each expression value has an equal probability of selection, and the time points are treated independently of one another. If not stated otherwise, the procedure was repeated 36 times, and we created 36 different resampled datasets (python script provided as Additional file 1). Each resampled dataset was analyzed by the ARSER algorithm with the stringency threshold (*q*-value) set to 0.05. HAYSTACK algorithm was used with the following parameter: p-value = 0.05; fold change = 2.0, correlation cutoff = 0.8; and background cutoff = 0.01. Using the oscillating transcripts detected the consensus between the 36 resampled datasets were calculated.

## Additional file

Additional file 1
**Models and simulations.** Python scripts used to generate time series, initial and resampled datasets are provided as ZIP-archive.
